# A Smartphone-Delivered Ecological Momentary Intervention for Problem Gambling (GamblingLess: Curb Your Urge): Single-Arm Acceptability and Feasibility Trial

**DOI:** 10.2196/25786

**Published:** 2021-03-26

**Authors:** Chloe O Hawker, Stephanie S Merkouris, George J Youssef, Nicki A Dowling

**Affiliations:** 1 Deakin University Geelong Australia; 2 Royal Children's Hospital Centre for Adolescent Health Murdoch Children’s Research Institute Melbourne Australia; 3 Melbourne Graduate School of Education University of Melbourne Parkville Australia

**Keywords:** gambling, craving, urge, self-efficacy, relapse, smartphone, self-help, treatment, ecological momentary assessment, ecological momentary intervention, mobile phone

## Abstract

**Background:**

Low uptake rates of traditional gambling treatments highlight the need for innovative treatment modalities. Smartphone apps can provide unprecedented access to real-time ecological momentary interventions (EMIs) delivered in people’s everyday lives.

**Objective:**

This study aims to examine the acceptability, feasibility, and preliminary effectiveness of GamblingLess: Curb Your Urge, the first smartphone app–delivered EMI that aims to prevent gambling episodes by reducing craving intensity in people seeking help for gambling problems.

**Methods:**

This study was a single-arm, 5-week acceptability and feasibility trial (1-week baseline and 4-week intervention periods) involving ecological momentary assessments (EMAs) delivered 3 times daily. The EMAs measured gambling episodes, cravings, and self-efficacy. Web-based evaluations at baseline, postintervention, and 1-month follow-up measured gambling outcomes (severity, cravings, frequency, expenditure, and self-efficacy) and the intervention’s perceived helpfulness, relevance, burden, satisfaction, and impact in relation to gambling cravings.

**Results:**

A total of 36 participants, of whom 22/36 (61%) were male and 34/36 (94%) were problem gamblers, completed the baseline measures, with 61% (22/36) completing the postintervention evaluation and 58% (21/36) completing the follow-up evaluation. The intervention was considered acceptable, as participants perceived all intervention content to be above average in helpfulness and the EMA to be highly relevant but somewhat burdensome. Participants reported that they were satisfied with the intervention and that the intervention improved their knowledge, attitudes, awareness, behavior change, intention to change, and help-seeking behavior for gambling cravings. Regarding the intervention’s feasibility, compliance rates for the EMA (51%) and EMI (15%) were low; however, the intervention was used 166 times, including 59 uses within 60 minutes of EMA completion and 107 on-demand uses. Regarding the intervention’s preliminary effectiveness, descriptive EMA data showed that, compared with the baseline period, 71% and 72% reductions in the average number of gambling episodes and craving occurrences were reported in the intervention period, respectively. In addition, clustered paired-sample two-tailed *t* tests revealed a significant 5.4% reduction in real-time craving intensity (*P*=.01) immediately after intervention use, which increased to 10.5% (*P*=.01), where use was recommended based on craving occurrence. At the group level, significant medium-to-large reductions were observed in mean gambling symptom severity (*P*=.01 and .003), cravings (*P*=.03 and .02), frequency (*P*=.01 and .004), and expenditure (*P*=.04 and .003) at postintervention and follow-up; moreover, increased mean gambling self-efficacy and craving self-efficacy (*P*=.01 and .01) were observed at postintervention and increased gambling self-efficacy (*P*=.04) was observed at follow-up. At the individual level, over a quarter of participants (6/22, 27% to 10/21, 48%) could be categorized as *recovered* or *improved* regarding their gambling symptom severity and cravings.

**Conclusions:**

The results support the acceptability, feasibility, and preliminary effectiveness of this app-delivered EMI for preventing gambling episodes through craving management in people with gambling problems, which has implications for extending the reach of evidence-based treatment to moments of vulnerability in people’s everyday lives.

## Introduction

### Background

Problem gambling is characterized by persistent and recurrent difficulties in limiting time and/or money spent gambling, which impacts the individual, their friends and family, and the community [1]. The harms associated with problem gambling are widespread, including financial difficulties, relationship conflict, emotional distress, health decrements, reduced productivity and job losses, and criminal activities [2]. Standardized prevalence rates estimate that 2.3% of the global population display past-year problem gambling. Australian rates estimate 0.4% to 0.6% for problem gambling, 1.9% to 3.7% for moderate-risk gambling, and 3% to 7.7% for low-risk gambling [3,4]. As such, problem gambling is a major public health issue with a burden of harm on par with depression and alcohol use disorders and almost twice that of substance use disorders, bipolar disorder, eating disorders, and schizophrenia combined [5].

### Treatment of Problem Gambling

Despite meta-analytic evidence indicating the efficacy of face-to-face delivered cognitive behavioral therapy (CBT) and motivational interviewing (MI) in gambling treatment [6-8], few people access face-to-face services (8%-16%) [9]. Commonly reported service barriers include personal reasons, such as shame, stigma, and a desire for self-management, and resource, geographic, and time constraints [9-12]. To overcome these barriers, novel treatment approaches that extend the reach of evidence-based treatment beyond the confines of face-to-face services are receiving increased attention. To date, such research has focused on web-based self-directed interventions, with a recent systematic review [13] demonstrating that the 2 available high-intensity, structured web-based interventions for problem gambling [14,15] were as effective as face-to-face services for reducing problem gambling severity, gambling frequency, and financial loss at posttreatment.

### Smartphone App–Delivered Ecological Momentary Interventions

Smartphone apps are a particularly advantageous modality for delivering self-directed interventions because of their ubiquitous use and extensive global networks that offer unprecedented treatment access [16,17]. As such, apps can serve as a “conduit for intervention any time and in almost any location” [18], which is considered important for preventing gambling behavior and relapse [19,20]. There is growing evidence for the acceptability, feasibility, and preliminary effectiveness of smartphone-delivered interventions for numerous chronic medical conditions [21-23] and mental health concerns [24,25]. Despite its promise, only a small number of apps delivering adjunctive [26,27] or stand-alone [28-30] interventions have been developed for problem gambling. Only 2 of these apps have undergone initial testing [26,27], with the results supporting their utility for increasing therapeutic homework completion [26] and reducing problem gambling severity [27]. Although these findings demonstrate the promise of app-delivered gambling interventions, they deliver static content that does not embrace the full potential of apps for delivering dynamic interventions when and where people need them.

Smartphone apps can deliver dynamic real-time interventions, termed *ecological momentary interventions* (EMIs), that aim to provide the right type and amount of in-the-moment support to people in their everyday lives [31]. To identify when an EMI is needed, apps can administer real-time *ecological momentary assessments* (EMAs) of relevant constructs at random or prespecified times. On the basis of the EMA results, apps can apply decision rules to determine the type, timing, and intensity of EMI delivered [31]. Smartphone app–delivered EMIs are increasingly employed in the broader mental health and addiction fields (eg, depression and anxiety [32], psychotic disorders [33], and alcohol and substance use [34-38]). Promisingly, addiction studies have demonstrated their preliminary effectiveness in identifying high-risk situations, reducing use and relapse incidence, and practicing real-time coping [34-37].

To the authors’ knowledge, however, there are only 2 smartphone app–delivered EMIs in the gambling field [39,40]. The first app, *Jeu-contrôle*, aims to support people to adhere to their gambling time and money limits by delivering personalized feedback about current gambling behavior compared with preset limits recorded during EMAs [39]; however, this app has not yet been evaluated. In contrast, the research team has developed and completed usability testing of GamblingLess: Curb Your Urge [40], the first smartphone-delivered EMI, tailored by responses to prompted EMA, for gambling craving management.

### GamblingLess: Curb Your Urge

GamblingLess: Curb Your Urge was developed as an app-delivered intervention within a suite of evidence-based web and mobile CBT and MI programs (GamblingLess) for problem gambling [41]. The intervention was designed to specifically target gambling cravings based on the relapse prevention model [42] and recent evidence that real-time cravings and self-efficacy to resist cravings were the strongest predictors of subsequent gambling behavior [43,44]. Ultimately, the intervention aims to prevent cravings from transitioning into gambling episodes by identifying when a consumer is experiencing a craving (via EMA) and subsequently providing strategies to manage their craving in the moment (via EMI). Similar to other app-delivered EMIs [18,34], the intervention provides a hybrid model, comprising a *push* intervention, whereby the app recommends an intervention at times of identified need, and a *pull* intervention, whereby consumers can access an intervention *on demand*, whenever and wherever they need it, without needing to complete an EMA [45]. The intervention was intended for use as a stand-alone or adjunctive treatment or relapse prevention tool.

Initial usability testing of the intervention with 29 key stakeholders, including past or current gamblers, gambling clinicians, and gambling researchers, promisingly revealed high ratings of expected helpfulness for managing cravings and usability in quantitative and qualitative assessments [40]. Testing of the intervention is now needed under its conditions of intended use, that is, in real-time, real-world contexts by people seeking help for gambling problems.

### Objectives

This study primarily aims to examine the acceptability and feasibility of GamblingLess: Curb Your Urge. The secondary aim of this study is to explore the intervention’s preliminary effectiveness, with the intervention hypothesized to (1) reduce real-time gambling craving intensity from immediately before to immediately after using the intervention and (2) reduce the preintervention levels of gambling symptom severity, gambling cravings, gambling frequency and expenditure, craving self-efficacy, and gambling self-efficacy at postintervention and 1-month follow-up evaluations.

## Methods

### Trial Design

This study was a single-arm, 5-week acceptability and feasibility trial (1-week baseline and 4-week intervention periods) involving EMAs delivered 3 times daily and web-based evaluations at baseline, postintervention, and a 1-month follow-up.

### Recruitment and Eligibility Criteria

The sample comprised 36 participants (14 women and 22 men) who completed the baseline measures, of whom 27 (82%) participants downloaded the app, 22 (61%) participants completed the postintervention evaluation, and 21 (58%) participants completed the follow-up evaluation. Individuals were eligible for inclusion if they were aged 18 years and above, resided in Australia, owned a smartphone, and were seeking help for their own gambling problem. Participants were permitted to access other help services throughout this study, with a mean number of 2.32 (SD 4.16) and 0.67 (SD 1.77) instances of additional professional help-seeking (eg, counseling, helpline, and general practitioners) reported by participants at the postintervention and follow-up evaluations, respectively. Participants were recruited across Australia via advertisements, using the tagline “Want to curb your gambling urges? And do it on your own? Then GamblingLess: Curb Your Urge may be for you,” posted in gaming venues and on the web (eg, paid and free social media advertisements) and media announcements (print, radio, and television). In addition, counselors from several Australian gambling treatment services provided information about the study to clients seeking additional support.

### The GamblingLess: Curb Your Urge Program

On the basis of the initial usability testing results [40], the research team refined the app intervention for this trial. Specifically, the intervention content was expanded to provide additional psychoeducation, mindfulness, and relaxation-based activities, and the tone was refined to be more normalizing. The intervention’s esthetics and level of engagement were also improved through the use of color and additional audiovisual components, which were subtitled to ensure that they could be completed anywhere and anytime.

In this trial, GamblingLess: Curb Your Urge consisted of a 4-week EMI delivered by an existing smartphone app platform (MetricWire). The intervention comprised 12 urge-curbing tips and activities (eg, *About My Urge*, *Delay and Distract*, and *Urge Surfing*) described in Table S1, with examples presented in Figure S1 in Multimedia Appendix 1. Each tip and activity took 1 to 5 minutes to complete.

#### EMA Feature

The app administered a brief EMA at random times during three prespecified periods (9 AM to noon, 1 PM-4 PM, and 5 PM-8 PM) via push notifications. The EMA comprised five core items to measure gambling episodes (since the last EMA), gambling cravings (current and since the last EMA), gambling self-efficacy (current confidence in ability to limit or stop gambling), and craving self-efficacy (current confidence in the ability to resist cravings) and up to an additional seven items depending on core item responses. These additional items were included to gain descriptive data about gambling cravings (eg, duration, frequency) that are largely absent in the corresponding empirical literature [43]. The EMA took approximately 1-2 minutes to complete, depending on the pattern of responses (the EMA items are given in Table S2 in Multimedia Appendix 1).

#### EMI Feature

The app’s EMI feature involved an automatic recommendation to use any urge-curbing tip or activity (Figure S2 in Multimedia Appendix 1) at the end of any EMA in which participants reported that they had a current craving (ie, responded *Yes* to EMA item 1; Table S2 in Multimedia Appendix 1). The program was also available 24×7 for use on demand. Whenever participants used a tip or activity (regardless of whether use was recommended or on demand), they were asked to rate the intensity of their craving to gamble from 0 (mild) to 10 (severe) immediately before and after using the tip or activity.

### Procedure

Study advertisements directed participants to the web-based baseline questionnaire, which was preceded by the provision of study information (eg, what participation involves and a description of the app intervention) followed by participant consent. In the baseline questionnaire, participants provided their contact details, which were used to email participants an instruction manual for downloading and using the app (eg, submitting EMAs and accessing the intervention content). Once enrolled, participants completed a 1-week EMA-only baseline period, followed by a 4-week intervention period (ie, EMA or EMI and 24×7 on-demand access to the intervention content). At the end of the intervention period, participants could no longer access the intervention. Participants were emailed a link to the web-based postintervention and 1-month follow-up questionnaires and, if required, were emailed up to 3 times to encourage questionnaire completion. Participants were recruited from September 2019 to June 2020. Participants were reimbursed up to Aus $60 (US $46.2) in e-gift vouchers, paid upon completion of the postintervention (Aus $30 [US $23.30]) and follow-up (Aus $30 [US $23.30]) evaluations. Reimbursement was not contingent on EMA completion. This study received ethics approval from the Deakin University Human Research Ethics Committee (Ethics ID: 2019-030).

### Measures

Baseline, postintervention, and follow-up evaluation measures were administered via structured web-based questionnaires hosted by Qualtrics (10 min to complete). During-intervention measures were available via the MetricWire app. An overview of the assessment measures and time points are given in Table S3 in Multimedia Appendix 1.

#### Acceptability Measures

Acceptability was assessed via a number of measures administered at postintervention, including (1) helpfulness of each urge-curbing tip or activity, and the relevance and burden of EMA items, via separate 11-point Visual Analogue Scales (VAS) from 0 (not helpful or relevant or burdensome) to 10 (very helpful or relevant or burdensome) [40]; (2) satisfaction with the intervention via the 3-item Client-Satisfaction Questionnaire-3 (CSQ-3; total score range 3-12) [46], with higher scores indicating greater overall satisfaction, participant needs met, and likelihood of future participant use; (3) impact of the intervention on participants’ awareness, knowledge, attitude, intention to change, help-seeking behavior, and behavior change in relation to gambling cravings via the 6-item App-Specific subscale of the Mobile App Rating Scale (MARS) [47], whereby the mean item score of 3 (range 1-5) indicates minimum acceptability [48]; and (4) a series of open-ended items assessing suggested improvements for any tip or activity that participants rated a 5 or less (out of 10) for helpfulness (*How could [tip/activity] be improved?*), any technical issues (*Please comment on any technical issues experienced.*), and general feedback about the app intervention (*Do you have any other comments or feedback?*) [40].

#### Feasibility Measures

Feasibility was assessed via participant recruitment at baseline and retention at postintervention and follow-up evaluations. Feasibility was also assessed using several app use metrics, including EMA compliance and EMI compliance. EMA compliance was measured as the rate at which participants completed the EMA during the baseline and intervention periods. EMI compliance was measured as the rate at which participants completed any intervention content within 60 minutes of receiving an EMI recommendation to use the intervention because they reported a current craving to gamble during an EMA [38,49,50]. Feasibility was also assessed via intervention use more generally. Upon inspection of the data, *any intervention use* was stratified by *EMA-prompted use* (defined as intervention use within 60 min of completing an EMA, regardless of whether they were recommended an activity based on craving occurrence) and *on-demand use* (defined as any other intervention use). The term *EMI use* was employed for the subset of EMA-prompted use in which use occurred following an EMI recommendation to use an intervention based on craving occurrence (note that this is equivalent to the EMI compliance rate).

#### Preliminary Effectiveness Measures

Preliminary effectiveness was assessed using changes in (1) real-time craving intensity measured by the aforementioned rating items administered immediately before and after using any urge-curbing tip or activity and (2) outcome measures completed at baseline, postintervention, and follow-up evaluations.

In the outcome evaluation, past-week gambling symptom severity (primary outcome) was measured using the 12-item Gambling-Symptom Assessment Scale (G-SAS) [51], with total scores (range 0-48) indicating mild (score of 8-20), moderate (score of 21-30), severe (score of 31-40), and extreme (score of 41-48) severity. In terms of secondary outcomes, gambling cravings were measured using the G-SAS Urge Subscale, which comprises the first 4 G-SAS items (score range 0-16) assessing the craving intensity, frequency, duration, and subjective control. Past-month gambling frequency (days) and expenditure (Aus $) on 6 gambling activities (electronic gaming machines or pokies, table games, racing, sports and event betting, number games, and informal private betting) were measured using a series of single items. Participants’ confidence in their ability to resist a craving to gamble (*craving self-efficacy*) and to limit or stop their gambling (*gambling self-efficacy*) were measured using separate 11-point VAS from 0 (not at all) to 10 (very confident) [43].

#### Descriptive and Diagnostic Measures

At baseline, participants reported their demographic information and whether they had a problem with 6 gambling activities (eg, table games and number games). Participants also completed the 9-item Problem Gambling Severity Index (PGSI) [52] to measure their past-year problem gambling status. Total PGSI scores (range 0-27) were used to indicate nonproblem (score of 0), low-risk (score of 1-2), moderate-risk (score of 3-7), and problem (score of 8 or higher) gambling.

### Statistical Analysis

Statistical analyses were conducted in Stata v13.0 [53]. Acceptability and feasibility were explored using descriptive statistics for quantitative variables (means and SDs for continuous variables and count and percentages for categorical data) and thematic content analysis at a semantic level for qualitative variables (ie, focusing on participant responses rather than latent meanings) [54]. Baseline differences in participants who did not complete a postintervention evaluation were calculated using chi-square tests with Fisher exact *P* values for categorical variables and *t* tests for continuous variables.

For the preliminary effectiveness evaluation using smartphone data, there were no missing data, as responses to all app-based items were required. To explore the intervention’s effectiveness for reducing real-time craving intensity, measured immediately before and immediately after intervention use via in-built rating items, a series of paired samples *t* tests using cluster-robust standard errors (to account for multiple intervention events clustered within individuals) assessed the change in mean intensity ratings. Separate *t* tests explored any intervention use and stratifications of EMA-prompted use (including EMI use) and on-demand use (defined in the Measures section). Given that some participants used multiple interventions in a short period because they could use the intervention as many times as they wanted, an additional *t* test assessed changes in craving intensity from immediately before the *first* intervention used to immediately after the *last* intervention used in any 60-minute window following an EMA. Owing to the pilot nature of this study and the small sample size, the unique effectiveness of each urge-curbing tip and activity was not examined.

For preliminary effectiveness outcome data collected at baseline, postintervention, and follow-up evaluations, there was no missingness across outcomes, except gambling expenditure (approximately 2.3% at baseline). Given the low amount of missing data and small sample size, analyses used a pairwise inclusion approach. To explore the intervention’s preliminary cumulative effectiveness over the study period, a series of paired samples *t* tests assessed group-level changes in mean outcome scores from baseline to postintervention and baseline to follow-up. Cohen *d* effect sizes were interpreted as small (0.20), medium (0.50), and large (0.80) [55]; Cohen *d* was not calculated for total gambling frequency and expenditure because of their skewed nature. Group-level examinations of effectiveness were also supplemented by 2 metrics of individual-level change in G-SAS gambling symptom severity, G-SAS gambling cravings, gambling frequency, gambling expenditure, craving self-efficacy, and gambling self-efficacy from baseline to postintervention and baseline to follow-up. First, a Reliable Change Index (RCI) [56] assessed change beyond that attributable to measurement error or chance, where RCI1.96 indicates reliable change with 95% confidence [57]. Second, clinically significant change was calculated using functional score ranges where possible (G-SAS score of 20 or less) or a convention of at least a 25% change in scores in the positive direction [58]. Four categories of change were created: *recovered* (the final score indicated a reliable change and was in the functional range), *improved* (the final score indicated a reliable change but was in the dysfunctional range), *unchanged* (the final score did not indicate a reliable change), or *deteriorated* (the final score indicated a reliable change in the negative direction).

## Results

### Sample Descriptive Statistics

Sample descriptive statistics for the 36 participants who completed the baseline measures are presented in Table 1. The majority of participants were men (22/36, 61%), in the age range of 35 to 49 years (17/36, 47%), worked full time (25/36, 69%), and used an iOS operating system (20/36, 56%). Majority of the participants identified having a problem with informal private betting (n=34; 94%) and electronic gaming machines (27/36, 75%). Almost all of the participants (34/36, 94%) met the PGSI criteria for problem gambling, with the remainder (2/36, 6%) displaying moderate-risk gambling.

**Table 1:** Descriptive statistics for the overall sample at baseline (N=36).

Demographic and diagnostic measures			Value, n (%)
Gender (male)			22 (61)
**Age group (years)**			
	18-24	1 (3)	
	25-34	14 (39)	
	35-49	17 (47)	
	50-64	4 (11)	
	65+	0 (0)	
Born in Australia			11 (31)
**Smartphone operating system**			
	Android	16 (44)	
	iOS (iPhone)	20 (56)	
**Employment**			
	Work full time	25 (69)	
	Work part time or casual	6 (17)	
	Unemployed	2 (6)	
	Full-time student	1 (3)	
	Full-time home duties	0 (0)	
	Retired	1 (3)	
	Other (work cover because of injury)	1 (3)	
**Past-month problem with gambling activities^a^**			
	Electronic gaming machines or pokies	27 (75)	
	Table games (eg, roulette and poker)	7 (19)	
	Horses, harness racing, or grayhound racing	15 (42)	
	Sports and event betting	14 (39)	
	Number games (eg, lotteries, keno, and bingo)	5 (14)	
	Informal private betting	34 (94)	
**Past-year problem gambling severity (Problem Gambling Severity Index)^b^**			
	No-risk gambling (score of 0)	0 (0)	
	Low-risk gambling (scores of 1-2)	0 (0)	
	Moderate-risk gambling (scores of 3-8)	2 (6)	
	Problem gambling (scores of 8 or higher)	34 (94)	
Hazardous alcohol use (Alcohol Use Disorders Identified Test-3)^c^			28 (78)
High psychological distress (Distress thermometer)^d^			29 (81)

^a^Participants could indicate a problem with more than one gambling activity.

^b^The Problem Gambling Severity Index was used, with scores ranging from 0 to 27.

^c^The Alcohol Use Disorders Identified Test-3 [59] was used to measure hazardous alcohol use, defined as a score of 1 or more (range 0-4).

^d^The Distress thermometer was used to measure psychological distress, defined as a score of 4 or more (range 0-10) [60].

### Acceptability

Descriptive acceptability statistics, presented in Table 2, were based on the 22 participants who completed the postintervention evaluation.

**Table 2:** Descriptive statistics for postintervention measures (n=22).

Acceptability measure		Value, mean (SD)
**Helpfulness ratings for “urge-curbing tips and activities”^a^**		
	Tip—Delay and Distract	7.41 (2.06)
	Tip—About My Urge	6.73 (2.39)
	Activity—Change Your Thoughts	6.55 (2.46)
	Activity—Get to Know Your Thoughts	6.55 (2.72)
	Activity—Urge Surfing	6.55 (2.79)
	Activity—Mindfulness	6.41 (2.52)
	Activity—Breathing Relaxation	6.23 (2.88)
	Tip—Tying it All Together	6.18 (2.56)
	Tip—Talk to Someone	6.18 (2.72)
	Activity—Progressive Muscle Relaxation	5.64 (2.66)
	Activity—Belly Breathing	5.55 (2.74)
	Activity—Brief Imagery	5.18 (2.89)
**Ecological momentary assessment items^a^**		
	Relevance of items	7.45 (2.02)
	Burdensome nature of items	4.59 (3.74)
**Satisfaction with intervention (CSQ-3^b^)**		
	Overall satisfaction with the intervention^c^	3.00 (0.69)
	The intervention met my needs^c^	2.82 (1.01)
	I would use the intervention again, if needed^c^	3.05 (0.79)
	Total satisfaction with intervention^d^	8.86 (2.05)
**Impact of the intervention (Mobile App Rating Scale app–specific subscale)^e^**		
	Awareness of the importance of addressing cravings	3.91 (1.15)
	Help seeking in future for cravings	3.82 (1.33)
	Knowledge and understanding of cravings	3.73 (1.16)
	Attitudes toward addressing cravings	3.68 (0.99)
	Intention to address cravings	3.64 (1.00)
	Behavior change: the app would help to manage cravings	3.64 (1.09)

^a^Mean scores can range from 0 to 10; tips and activities are presented in descending order from highest to lowest mean helpfulness rating.

^b^CSQ-3: Client-Satisfaction Questionnaire-3.

^c^The mean CSQ-3 item scores ranged from 1 to 4.

^d^The mean CSQ-3 total scores ranged from 3 to 12.

^e^The mean Mobile App Rating Scale scores can range from 1 to 5; items are presented in descending order, from the highest to lowest mean rating.

#### Intervention Helpfulness and EMA Relevance and Burden

The mean ratings for the perceived helpfulness of all urge-curbing tips and activities were higher than 5 out of 10, indicating above-average helpfulness. The highest rated overall were Delay and Distract (mean 7.41, SD 2.06), About My Urge (mean 6.73, SD 2.39), Change Your Thoughts (mean 6.55, SD 2.46), Get to Know Your Thoughts (mean 6.55, SD 2.72), and Urge Surfing (mean 6.55, SD 2.79). The mean ratings of the EMA’s perceived relevance were high (7.45 out of 10, SD 2.02) and burdensome nature were average (4.59 out of 10, SD 3.74).

#### Satisfaction With the Intervention

On the CSQ-3, participants scored an average of 8.86 out of 12 (SD 2.05). Individual item mean scores of approximately 3 out of 4 indicated that participants were mostly satisfied with the intervention, that the intervention met most of their needs, and that they would likely use it again to manage their cravings.

#### Impact of the Intervention

Mean scores across the MARS app–specific subscale items (range 3.64-3.91 out of 5) met acceptability standards (score of 3). From highest to lowest rated impact, participants indicated that the intervention improved their awareness about the importance of addressing cravings, future help seeking for cravings, knowledge and understanding of cravings, attitudes toward addressing cravings, and intention to address and ability to manage cravings.

#### Suggested Improvements

Overall, 16 participants rated the helpfulness of at least one tip or activity a 5 or below (out of 10) for managing cravings and were subsequently asked to suggest improvements on that tip or activity. Most of these participants tended to give a variation of “unsure” or no improvements required. Nevertheless, 1 participant suggested that Delay and Distract, Mindfulness, Urge Surfing, and Change Your Thoughts could be improved by providing more examples of “what has worked for others”. Furthermore, 1 participant suggested improving Talk to Someone by adding a live chat function to enable in-the-moment messaging support and 2 participants suggested improving the mindfulness- and relaxation-based activities by linking them to “a proper meditation/relaxation program*”* and incorporating additional audiovisual components.

#### Technical Issues

The majority of participants (15/22, 68%) reported no technical issues with the intervention. Five participants reported that the audiovisual content was slow to load, 1 participant reported that the app exceeded their phone’s storage capacity, and 1 participant reported that the tips and activities incorrectly appeared as *not completed* after completion. Almost all of the participants (20/22, 91%) reported no EMA technical issues; however, 1 participant reported that they did not receive notifications and 1 participant reported that EMAs wrongly displayed as *not completed* after completion.

#### General Feedback

Half of the participants (11/22, 50%) provided general feedback about the intervention, with most of them (7/22, 32%) providing positive feedback. Specifically, 3 participants found the EMA notifications helpful daily reminders to *stay on track and not gamble* and 4 participants found the intervention content to be highly accessible and helpful, with 1 participant stating that the app was “a great way to have different tools available at any time” and another stating that “the app worked well, was easy to use, notifications were good, (and the) training things were helpful and informative.” Four participants provided negative feedback about the EMA notifications, whereby 2 participants requested tailoring capabilities to user-specified times (eg, “pay day”), 1 participant stated that the notifications “reminded me about gambling when I wasn’t thinking about it,” and 1 participant thought that the notifications were too frequent.

### Feasibility

Consistent with the acceptability statistics, the feasibility statistics are based on the 22 participants who completed the postintervention evaluation, with the exception of the *Recruitment and Retention* section that details participant numbers throughout the study.

#### Recruitment and Retention

Over the 1-month recruitment period, 56 gamblers consented to participate in this study. Of these, 36 gamblers completed the baseline measures, 14 gamblers did not complete any baseline measures, and 6 gamblers completed only the baseline demographic measures. Of the 36 participants who completed the baseline measures, 9 participants did not download the app, 1 participant downloaded the app but did not use it, and 4 participants did not use the app beyond the first week of the study and did not complete the postintervention evaluation. The remaining 22 participants used the app and completed the postintervention evaluation, with 21 of these participants completing the 1-month follow-up evaluation. Of the 36 participants who completed baseline measures, 61% (22/36) completed postintervention evaluations and 58% (21/36) completed follow-up evaluations. There were no significant differences in any baseline variable between participants who did or did not complete the postintervention evaluation.

#### EMA Compliance

The EMAs had a compliance rate of 68% in the baseline period (mean 14.27, SD 5.68 out of 21 EMAs), 47% in the intervention period (mean 54.05, SD 33.37 out of 84 EMAs), and 51% overall (mean 54.05, SD 33.37 out of 105 EMAs).

#### EMI Compliance

The EMI compliance rate was 15%, as participants used an intervention 13 out of the 87 times that they were recommended to do so based on craving occurrence.

#### Intervention Use

Overall, 19 participants used the intervention at least once during the intervention period. Table 3 presents the descriptive statistics for intervention use. The intervention was used a total of 166 times (median 7; range 1-33), including 59 EMA-prompted uses (defined as intervention use within 60 min of completing an EMA), of which 13 were EMI uses (defined as intervention use within 60 min of completing an EMA and following an EMI recommendation to use an intervention because the participant reported a current craving). Of the 59 EMA-prompted uses, participants used the intervention once between EMAs on 29 occasions, 2 times on 10 occasions, 3 times on 2 occasions, and 4 times on 1 occasion. In contrast, there were 107 on-demand uses (defined as any other intervention use). The most used intervention content included About My Urge (22 uses), Talk to Someone (19 uses), and Mindfulness (18 uses).

**Table 3:** Total intervention use frequencies (count and percentages) stratified by ecological momentary assessment–prompted and on-demand use during the 4-week intervention period (n=22).

Intervention content	Total use, n (%)	Ecological momentary assessment–prompted use^a^, n (%)	On-demand use^b^, n (%)
Tip—About My Urge	22 (13.3)	7 (11.9)	15 (14.0)
Tip—Talk to Someone	19 (11.4)	9 (15.3)	10 (9.3)
Activity—Mindfulness	18 (10.8)	7 (11.9)	11 (10.3)
Activity—Get to Know Your Thoughts	16 (9.6)	4 (6.8)	12 (11.2)
Activity—Change Your Thoughts	13 (7.8)	1 (1.7)	12 (11.2)
Tip—Tying it All Together	13 (7.8)	6 (10.2)	7 (6.5)
Activity—Belly Breathing	12 (7.2)	8 (13.6)	4 (3.7)
Activity—Urge Surfing	12 (7.2)	2 (3.4)	10 (9.3)
Activity—Brief Imagery	11 (6.6)	4 (6.8)	7 (6.5)
Tip—Delay and Distract	11 (6.6)	5 (8.5)	6 (5.6)
Activity—Breathing Relaxation	10 (6.0)	3 (5.1)	7 (6.5)
Activity—Progressive Muscle Relaxation	9 (5.4)	3 (5.1)	6 (5.6)
Total	166 (100.0)	59 (100.0)	107 (100.0)

^a^Ecological momentary assessment–prompted use is defined as intervention use within 60 min of completing an ecological momentary assessment, regardless of whether participants were recommended an activity based on craving occurrence.

^b^On-demand use is defined as any other intervention use.

### Preliminary Effectiveness

#### Descriptive Statistics

On the basis of the 22 participants who completed the postintervention evaluation, Table 4 presents descriptive statistics for EMA outcome variables. Compared with the 1-week baseline period, there was a 71% reduction in the average number of gambling episodes and a 72% reduction in the average number of craving occurrences, as reported in the 4-week intervention period. Furthermore, craving self-efficacy and gambling self-efficacy increased from mean ratings of 5.85 (SD 2.95) and 4.82 (SD 3.01) out of 10 (where 10=complete confidence in ability to resist a craving and to limit or stop gambling) over the baseline period to mean ratings of 6.63 (SD 2.61) and 5.72 (SD 3.02) over the intervention period, respectively.

**Table 4:** Descriptive statistics of ecological momentary assessment outcome variables (n=22).

Variable		One-week baseline period	Four-week intervention period	Total
**Gambling episodes, n (%)**				
	No	304 (86.1)	835 (93.6)	1139 (91.5)
	Yes	49 (13.9)	57 (6.4)	106 (8.5)
**Gambling episode win or loss status, n (%)**				
	Win	10 (20.4)	8 (14.0)	18 (17.0)
	Loss	33 (67.3)	42 (73.7)	75 (70.7)
	Broke even	6 (12.2)	7 (12.3)	13 (12.3)
**Gambling episode win amount, Aus $ (US $), n (%)**				
	1-150 (0.70-115.6)	6 (60.0)	2 (25.0)	8 (44.4)
	151-500 (116.4-385.3)	2 (20.0)	2 (25.0)	4 (22.2)
	501-1000 (386.1-770.6)	1 (10.0)	3 (37.5)	4 (22.2)
	1001-7500 (771.3-5779.2)	1 (10.0)	1 (12.5)	2 (11.1)
**Gambling episode loss amount, Aus $ (US $), n (%)**				
	1-150 (0.70-115.6)	9 (27.3)	12 (28.6)	21 (28.0)
	151-500 (116.4-385.3)	15 (45.4)	7 (16.7)	22 (29.3)
	501-1000 (386.1-770.6)	5 (15.2)	15 (35.7)	20 (26.7)
	1001-7500 (771.3-5779.2)	4 (12.1)	8 (19.0)	12 (16.0)
**Craving occurrences, n (%)**				
	No	274 (77.6)	805 (90.2)	1079 (86.7)
	Yes	79 (22.4)	87 (9.8)	166 (13.3)
Craving intensity^a^, mean (SD)		6.35 (3)	6.59 (3)	6.47 (3)
Craving frequency^b^, mean (SD)		4.22 (9)	5.39 (16)	4.78 (13)
Craving duration (min)^c^, mean (SD)		36.64 (59)	50.65 (79)	43.36 (70)
Subjective control over cravings^a^, mean (SD)		5.96 (4)	5.95 (4)	5.96 (4)
Craving self-efficacy^a^, mean (SD)		5.85 (3)	6.63 (3)	6.41 (3)
Gambling self-efficacy^a^, mean (SD)		4.82 (3)	5.71 (3)	5.46 (3)

^a^Range 0-10 on a Visual Analogue Scale.

^b^Range 1-180 craving occurrences.

^c^Range 0-480 minutes.

#### Real-Time Reduction in Craving Intensity Immediately After Intervention Use

On the basis of the 22 participants who completed the postintervention evaluation, the results of clustered paired-sample *t* tests revealed a significant decrease of 5.4% in momentary craving intensity from immediately before to immediately after *any intervention use* (*P*=.01), with a medium effect (Cohen *d*_z_=−0.64; 95% CI −1.13 to −0.14). The results demonstrated a 7.5% decrease in craving intensity for EMA-prompted use (*P*=.03; Cohen *d*_z_=−0.72; 95% CI −1.34 to −0.06), and more specifically, a 10.5% decrease for EMI use (*P*=.01; Cohen *d*_z_=−1.29; 95% CI −2.48 to −0.03). In contrast, there was a 4.5% decrease for on-demand use (*P*=.01; Cohen *d*_z_=−0.66; 95% CI −1.16 to −0.14). There was no added benefit of using multiple interventions between EMAs, as the results demonstrated an 8.6% decrease in craving intensity after one use (*P*=.02; Cohen *d*_z_=−0.94; 95% CI −1.67 to −0.17) compared with a 7.7% decrease after more than one use (*P*=.29; Cohen *d*_z_=−0.63; 95% CI −1.49 to 0.28).

#### Change in Gambling Symptom Severity, Cravings, Frequency, Expenditure, and Self-Efficacy at Postintervention and Follow-Up

Descriptive statistics for gambling symptom severity, cravings, frequency, expenditure, and self-efficacy measured at baseline (n=36), postintervention (n=22), and follow-up (n=21) are presented in Table 5. At the group level, participants showed significant reductions in mean G-SAS gambling symptom severity (*d*=0.61 and 0.75; *P*=.01 and .003), G-SAS cravings (*d*=0.49 and 0.55; *P*=.03 and .02), gambling frequency (*P*=.01 and .004), and gambling expenditure (*P*=.04 and .003) from baseline to postintervention and follow-up, respectively. In addition, participants showed a significant increase in mean craving self-efficacy and gambling self-efficacy from baseline to postintervention (*d*=0.66 and 0.60; *P*=.01 and .01) and gambling self-efficacy (*d*=0.49; *P*=.04) but not craving self-efficacy (*P=*.20) from baseline to follow-up.

**Table 5:** Descriptive statistics (mean and SD) for measures administered at baseline, postintervention, and 1-month follow-up evaluations.

Outcome measure			Baseline (N=36)		Postintervention (n=22)		One-month follow-up (n=21)
**Past-week gambling symptom severity^a^ (Gambling-Symptom Assessment Scale), mean (SD)**			30.92 (8)		22.18 (10)		20.43 (11)
	Mild, n (%)	5 (14)		9 (41)		11 (52)	
	Moderate, n (%)	11 (31)		7 (32)		6 (29)	
	Severe, n (%)	14 (39)		6 (27)		4 (19)	
	Extreme, n (%)	6 (17)		0 (0)		0 (0)	
Past-week gambling cravings^b^ (Gambling-Symptom Assessment Scale-Urge Subscale), mean (SD)			9.81 (3)		6.82 (3)		6.57 (4)
Total gambling frequency (days) in the last month, mean (SD)			14.22 (13)		5.27 (6)		3.50 (5)
Total gambling expenditure (Aus $) in the last month, mean (SD)			2894.17 (3736)		1675.68 (2484)		629.48 (1232)
Current gambling self-efficacy^c^, mean (SD)			3.97 (3)		6.32 (3)		6.00 (3)
Current craving self-efficacy^c^, mean (SD)			4.72 (3)		6.77 (2)		6.00 (3)
**Gambling treatment goal, n (%)**							
	Quit gambling altogether	24 (67)		14 (64)		14 (67)	
	Quit gambling activities I think I have a problem with	8 (22)		5 (23)		5 (24)	
	Cut back gambling activities I think I have a problem with	4 (11)		3 (14)		2 (10)	
Additional professional help-seeking for gambling problems (number of times) in the last month, mean (SD)			N/A^d^		2.32 (4)		0.67 (2)

^a^Gambling-Symptom Assessment Scale scores can range from 0 to 48, categorized as mild (score of 8-20), moderate (score of 21-30), severe (score of 31-40), and extreme (score of 41-48).

^b^Gambling-Symptom Assessment Scale-Urge Subscale scores can range from 0 to 16.

^c^Self-efficacy scores can range from 0 to 10 on a Visual Analogue Scale.

^d^N/A: not applicable.

Table 6 presents clinically significant change on these measures from baseline to postintervention and follow-up. At an individual level, approximately a quarter of participants were *recovered* at postintervention and follow-up, respectively, on G-SAS gambling symptom severity (6/22, 27%; 6/21, 29%) and G-SAS gambling cravings (6/22, 27%; 7/21, 33%), the majority were *unchanged* (symptom severity: 13/22, 59%; 11/21, 52%; cravings: 14/22, 64%; 14/21, 67%), and a small number of participants were *deteriorated* at postintervention (symptom severity: 1/22, 5%; cravings: 2/21, 9%); however, none were deteriorated at follow-up. The majority of participants were *unchanged* at postintervention and follow-up on gambling frequency (20/22, 91%; 18/21, 90%), gambling expenditure (19/22, 86%; 17/21, 81%), craving self-efficacy (18/22, 82%; 17/21, 81%), and gambling self-efficacy (18/22, 82%; 18/21, 86%); however, none were *deteriorated* on these measures.

**Table 6:** Clinically significant changes in outcome measures at postintervention (n=22) and 1-month follow-up (n=21) evaluations.

Outcome measure		Postintervention (n=22)	One-month follow-up (n=21)
**Gambling symptom severity (G-SAS^a^), n (%)**			
	Recovered^b^	6 (27)	6 (28)
	Improved	2 (9)	4 (19)
	Unchanged	13 (59)	11 (52)
	Deteriorated	1 (4)	0 (0)
**Gambling cravings (G-SAS Urge Subscale), n (%)**			
	Recovered	6 (27)	7 (33)
	Improved	0 (0)	0 (0)
	Unchanged	14 (63)	14 (66)
	Deteriorated	2 (9)	0 (0)
**Total gambling frequency^c^ (days), n (%)**			
	Recovered	2 (9)	2 (10)
	Improved	0 (0)	0 (0)
	Unchanged	20 (90)	18 (90)
	Deteriorated	0 (0)	0 (0)
**Total gambling expenditure (Aus $), n (%)**			
	Recovered	3 (13)	4 (19)
	Improved	0 (0)	0 (0)
	Unchanged	19 (86)	17 (80)
	Deteriorated	0 (0)	0 (0)
**Craving self-efficacy (11-point VAS^d^), n (%)**			
	Recovered	0 (0)	0 (0)
	Improved	4 (18)	3 (14)
	Unchanged	18 (81)	17 (80)
	Deteriorated	0 (0)	1 (4)
**Gambling self-efficacy (11-point VAS), n (%)**			
	Recovered	0 (0)	0 (0)
	Improved	4 (18)	3 (14)
	Unchanged	18 (81)	18 (85)
	Deteriorated	0 (0)	0 (0)

^a^G-SAS: Gambling-Symptom Assessment Scale.

^b^Recovered: the final score indicated a reliable change and was in the functional range, indicated by a score of 20 or less on the Gambling-Symptom Assessment Scale or at least a 25% reduction in scores for gambling cravings, total gambling frequency, and total gambling expenditure, and at least a 25% increase in scores for craving self-efficacy and gambling self-efficacy, in postintervention and follow-up evaluations.

^c^n=20 at 1-month follow-up, as 1 participant did not report gambling frequency.

^d^VAS: Visual Analogue Scale.

## Discussion

### Principal Findings

The results of this study demonstrate the acceptability, feasibility, and preliminary effectiveness of the first smartphone app–delivered EMI (GamblingLess: Curb Your Urge), which aims to prevent gambling episodes through reduced craving intensity in people seeking help for gambling problems.

#### Acceptability

Overall, the app intervention was rated favorably across quantitative and qualitative measures of acceptability. Participants indicated that they were mostly satisfied with the intervention, as it met most of their needs and could be used to manage their cravings in the future, based on mean CSQ-3 scores. Furthermore, participants indicated that the intervention improved their awareness, knowledge, attitude, and intentions to address cravings and, importantly, their ability to manage cravings, based on mean MARS item scores. Qualitatively, the majority of participants provided positive feedback about the intervention, particularly in relation to its accessibility, helpfulness, and effectiveness in preventing gambling episodes.

Promisingly, participants rated all of the urge-curbing tips and activities as above average for helpfulness in managing cravings. Consistent with the evidence base for gambling treatments, in which CBT techniques are considered best practice [7,41,61,62], the highest rated content involved CBT techniques, including psychoeducation (About My Urge); cognitive reappraisal (Get to Know Your Thoughts and Change Your Thoughts); and relapse prevention techniques, including distraction (Delay and Distract) and a mindfulness practice to *surf* the wave of a craving without acting on it (Urge Surfing). Participants suggested that this content could be further improved by providing more concrete examples of skill implementation. The lowest rated content included relaxation-based or imaginal techniques (Progressive Muscle Relaxation, Belly Breathing, and Brief Imagery), with 2 participants suggesting their improvement via linkage with related programs outside of the app. Taken together, these findings suggest that intervention content variability and examples of skill implementation are important for people to find, and learn to apply, what works best for them.

With respect to the EMA, participants considered them highly relevant but somewhat burdensome, which represents an improvement from the initial usability testing [40] in which they were rated in the average range on both domains. To reduce its level of burden, the frequency of EMAs could be reduced or, as 2 participants suggested, made personally customizable to specific times (eg, *pay day*). Indeed, the combined results of usability testing and this trial indicated that tailoring capabilities were considered particularly important for consumers to enable personalized check-ins and treatment.

#### Feasibility

Recruitment and retention have posed a challenge in studies examining app-delivered interventions for mental health [61], particularly for problem gambling [29]. Despite extensive recruitment efforts, only 36 people completed baseline measures, with approximately 60% of these people completing postintervention (n=22) and follow-up (n=21) evaluations. Retention rates in this trial were lower than those of other feasibility trials of app-delivered EMIs for alcohol and substance use, where rates range from 63% to 90% [35,38]. Although not directly comparable, retention was on par with the only other feasibility trial of a gambling intervention app (60%) [26]. Future research may benefit from examining consumer profiles to enable targeted recruitment campaigns and tailored interventions to particular demographic groups, the latter of which may also facilitate trial retention through improved engagement with the app [62].

In this trial, the feasibility was measured via compliance with the EMA and EMI features. The overall compliance was 51% for EMAs and 15% for EMIs. Although there are no directly comparable gambling app intervention studies, previous gambling EMA studies have reported variable compliance rates from 50% to over 90% [43,63]; however, notably, EMA completion was financially incentivized in these studies. As financial incentives are not feasible for ongoing app interventions in real-world contexts, it would be useful to examine ways to improve EMA compliance. In addition, EMA compliance may have been reduced in this study as participants could access intervention content *on demand* without needing to complete a more time-consuming EMA. While this is a limitation of providing a hybrid *push or pull* intervention, this approach supports consumers to access interventions regardless of their awareness of vulnerable states or motivation to seek support [45]. Furthermore, EMA compliance may improve outside of a research context, as it is envisioned that the EMA feature of future iterations of the intervention for real-world use would be briefer (eg, to only measure current craving occurrence) and customizable regarding the frequency and timing of administration (eg, to user-specified times). The EMI compliance rate in this study was also low, however, as participants used the intervention only 13 of the 87 times that they were recommended to do so. Low EMI compliance may reflect the limitations of the app platform, whereby intervention content cannot be automatically delivered (rather than just recommended). Although this limitation enabled a more nuanced evaluation of the intervention’s preliminary effectiveness under different conditions of use, future app iterations may benefit from using platforms that can automatically deliver an EMI at times of need.

Across the 4-week intervention period, participants used the intervention a total of 166 times (median 7 uses; range 1-33), including 59 EMA-prompted uses and 107 on-demand uses. This rate of intervention uptake (7 uses over 4 weeks) appears comparable with that of traditional face-to-face gambling services, in which treatment typically involves 1 session per week for 10 weeks [64]. Despite similar uptake rates, we might expect higher app use given its 24×7 availability and evidence of EMI underutilization. Although apps can overcome a number of barriers to accessing face-to-face services (eg, geographic constraints) [11,12], future research should explore app-specific barriers to use at times of identified need. The higher rate of on demand (*pull*) intervention use compared with EMA-prompted and EMI (*push*) use suggests either that the EMA item measuring craving occurrence was not adequately identifying the times of vulnerability and/or that participants knew when they needed to access an intervention and were motivated to do so [45]. To address the latter possibility, future app interventions may benefit from incorporating additional user-initiated EMAs [18,34], whereby participants can record a gambling craving or episode at any time in the app.

#### Preliminary Effectiveness

A unique strength of EMA and EMI data is the capability to explore real-time intervention effects in people’s everyday lives. Promisingly, descriptive EMA data showed that, compared with the baseline period, there was an approximately 71% to 72% reduction in the average number of gambling episodes and craving occurrences and an approximately 1-point increase in the mean craving self-efficacy and gambling self-efficacy on an 11-point VAS, reported in the intervention period. In addition, the results showed that the intervention led to a medium decrease of 5.4% in real-time craving intensity from immediately before to immediately after use. This effect increased with a large decrease of 10.5% for EMI use (ie, where participants had a current craving to gamble) and 7.5% for EMA-prompted use (ie, use within 60 min of an EMA regardless of whether participants had a craving) and reduced with a medium decrease of 4.5% for on-demand use (ie, any other intervention use), which intuitively supports the intervention’s increased effectiveness in moments of vulnerability. The results also suggested an optimal dosage of 1 intervention between EMAs, as there was no added benefit of multiple uses.

Given the small sample size and limited power to detect significant effects, this study also explored the intervention’s preliminary effectiveness at both the group and individual level. At the group level, participants displayed significant medium-to-large reductions in mean gambling symptom severity and cravings and reductions in gambling frequency and expenditure at postintervention and 1-month follow-up evaluations. Participants also displayed significant medium-to-large increases in gambling self-efficacy at postintervention and follow-up and craving self-efficacy at postintervention. At an individual level, more than a quarter of participants were considered *recovered* or *improved* on G-SAS gambling symptom severity (8/22, 36%; 10/21, 48%) and G-SAS gambling cravings (6/22, 27%; 7/21, 33%), at postintervention and follow-up respectively, with the majority remaining *unchanged*, which compares with more than a half of participants (*n*=55, 64%) on gambling symptom severity in a randomized trial of its *parent* program (GamblingLess) [41]. A small proportion of participants in this study were also *recovered* or *improved* on gambling frequency, expenditure, gambling self-efficacy, and craving self-efficacy (2/22, 9% to 4/22, 19%). By the end of this study, only 1 participant was considered *deteriorated* on craving self-efficacy but no other measure. Although it is possible that these results reflect a highly motivated sample, natural recovery, or recovery resulting from concurrent treatment, these findings provide preliminary evidence for the effectiveness of the intervention, which would likely increase further when combined with a fuller suite of gambling interventions.

### Strengths and Limitations

This study has several important strengths. First, the intervention was based on sound theory [42] and empirical research [43] to provide an evidence-based, app-delivered gambling intervention that supports self-management in light of low professional treatment uptake rates [9]. Second, this study and a preceding usability study [40] demonstrated the value of including consumers’ feedback in the early stages of intervention development and evaluation, which is integral to short- and long-term app engagement [21,65]. Finally, the use of smartphones in this study to deliver real-time interventions to people in their natural environments and the lack of financial incentives for EMA or EMI completion support real-world uptake of the app.

The study findings should be interpreted in light of several limitations. Although this study’s sample size was consistent with other acceptability and feasibility trials [18,26,38], it was relatively small, which limits the generalizability of the results and power to detect significant effects. Future studies would benefit from using a larger sample, which may require a longer recruitment period and substantial recruitment efforts [29]. Somewhat low rates of EMA and EMI compliance and intervention use also limited our statistical power to detect significant effects. Consistent with participant acceptability feedback, these rates could be increased by improving the intervention’s level of engagement, such as through increased content variability and customizable features [62]. In addition, preliminary evidence of the effectiveness of the intervention may have been inflated by assessment reactivity and demand characteristics, as participants could likely ascertain the research aims. Interestingly, a recent gambling EMA study found a weak reactivity effect in the opposite direction than expected, as participants reported more gambling activity throughout the study [44], which is consistent with the broader addiction literature, which did not indicate strong reactivity effects [66]. Nevertheless, future studies may improve the generalizability of their results by examining intervention effectiveness using publicly available, routinely collected data from app use in the real world [67]. Finally, it is acknowledged that this study did not redress the dearth of economic data to support the use of smartphone-delivered interventions in mental health treatment, which future feasibility studies ought to consider [68].

### Conclusions

The findings of this study support the acceptability, feasibility, and preliminary effectiveness of GamblingLess: Curb Your Urge, the first app-delivered EMI for craving management in people seeking help for gambling problems. These findings indicate the utility of developing targeted, real-time app interventions for problem gambling, particularly as an offshoot of more comprehensive programs with a developing or established evidence base, such as GamblingLess [41]. In so doing, app interventions may extend the reach of evidence-based treatment beyond the confines of face-to-face services to moments of need in people’s everyday lives.

## Appendix

Multimedia Appendix 1 GamblingLess: Curb Your Urge: Intervention content, ecological momentary assessment items, an overview of evaluation measures, and the ecological momentary intervention feature. EMA: ecological momentary assessment; EMI: ecological momentary intervention.

## Abbreviations

CBT: cognitive behavioral therapy
CSQ-3: Client-Satisfaction Questionnaire-3
EMA: ecological momentary assessment
EMI: ecological momentary intervention
G-SAS: Gambling-Symptom Assessment Scale
MARS: Mobile App Rating Scale
MI: motivational interviewing
PGSI: Problem Gambling Severity Index
RCI: Reliable Change Index
VAS: Visual Analogue Scale
