# Supplementary material for: A Smartphone-Delivered Ecological Momentary Intervention for Problem Gambling (GamblingLess: Curb Your Urge): Single-Arm Acceptability and Feasibility Trial
Source: J Med Internet Res. 2021 Mar 26;23(3):e25786. doi: 10.2196/25786 (PMC8088874; doi:10.2196/25786)
Supplement: Multimedia Appendix 1 [file jmir_v23i3e25786_app1.docx]

# **Supplementary Material**

# GamblingLess: Curb Your Urge: An acceptability and feasibility trial of a smartphone-delivered ecological momentary intervention for problem gambling

**Author List:** Hawker, C. O.^1^, Merkouris, S. S.^1^, Youssef, G. J.^1, 2^ & Dowling, N. A.^1, 3*^

**Author affiliations:**

1 School of Psychology, Deakin University, Geelong, Australia.

2 Centre for Adolescent Health, Murdoch Children’s Research Institute, Royal Children's Hospital, Melbourne, Australia

3 Melbourne Graduate School of Education, University of Melbourne, Parkville, Australia.

* Corresponding author: A/Prof Nicki Dowling, School of Psychology, Deakin University, email: [nicki.dowling@deakin.edu.au](mailto:nicki.dowling@deakin.edu.au), phone: +61 3 9244 5610

### Table S1

*GamblingLess: Curb Your Urge: Intervention Content*

| **‘Urge Curbing Tips and Activities’** | **Techniques employed within activities** |
| --- | --- |
|  |  |
| 1. Tip – About My Urge | Psychoeducation about the nature of cravings (analogous to a wave) and the impact of acting on cravings for increasing the intensity and frequency of future cravings |
| 2. Tip – Delay and Distract | Interference-based technique encouraging consumers to delay the decision to act on their craving by distracting themselves in the short-term |
| 3. Tip – Talk to Someone | Encouraged seeking support for managing cravings by talking to someone (e.g., friend or professional) face-to-face or via telephone or online services. Provided contact information for various gambling services (e.g., Gamblers Helpline) |
| 4. Activity – Belly Breathing | Relaxation-based technique to engage in belly breathing to reduce excitement or tension during a craving. Provided a 3-minute guided video. |
| 5. Activity – Breathing Relaxation | Relaxation-based technique using ‘box breathing’ to reduce excitement or tension during a craving. Provided a 4-minute guided video. |
| 6. Activity – Mindfulness | Psychoeducation about, and suggestions for ways to practice, mindfulness in consumers’ everyday lives. Provided a 5-minute guided mindfulness video and suggestions for other apps to try (e.g., Smiling Mind). |
| 7. Activity – Progressive Muscle Relaxation | Mindfulness technique to tense and relax particular muscles to reduce excitement and tension during a craving. Provided a 10-minute video (divided into two parts) for upper and lower body relaxation. |
| 8. Activity – Urge Surfing | Mindfulness and relapse prevention technique to imagine ‘riding the wave’ of a craving without acting on it. Provided an instructional image and 3-minute guided video. |
| 9. Activity – Brief Imagery | Imaginal exposure technique that provides several prompts to encourage not acting on cravings. Provided a 4-minute instructional video. |
| 10. Activity – Get to Know Your Thoughts | Psychoeducation and CBT technique to identify craving-related thoughts at different stages of the craving ‘wave’. Provided a 5-minute instructional video. |
| 11. Activity – Change Your Thoughts | CBT technique to re-appraise craving-related thoughts, such as by weighing the costs and benefits of acting on a craving in the short- and long-term. |
| 12. Tip – Tying it All Together | Relapse prevention technique, in which the key learnings from all of the other tips and activities are summarised to remind consumers of all of the ways to manage cravings. |

### Table S2

*GamblingLess: Curb Your Urge: EMA items*

| **EMA items** | **Construct** | **Response Options** |
| --- | --- | --- |
|  |  |  |
| 1. Are you having an urge to gamble right now?^a^ | Craving occurrence | 1. Yes/No |
| - 1. How strong is your urge (from 0 – mild to 10 – severe)? | Craving intensity | a. Rating 0-10 |
| 1. Have you had an urge to gamble since the last notification (not including right now)?^a^ | Craving occurrence | 1. Yes/No |
| - 1. How many times did you experience urges to gamble? | Craving frequency | a. Open text |
| - 1. How many minutes, in total, were you preoccupied with your urges to gamble? | Craving duration | b. Open text |
| - 1. How strong were your urges, on average (from 0 – mild to 10 – severe)? | Craving intensity | c. Rating 0-10 |
| - 1. How much were you able to control your urges (from 0 – no control to 10 – complete control)? | Subjective control over cravings | d. Rating 0-10 |
| 1. Have you gambled on your problem form (e.g., pokies) since the last notification (not including right now)?^a^ | Gambling episodes | 4. Yes/No |
| 1. Did you win, lose, or break even? | Gambling win/loss status | a. Win/Lose/ Break Even |
| - 1. How much did you [win/lose] in total? | Gambling win/loss amount | b. Response options from $1 to $10,000+ |
| 1. Right now, how confident are you that you would be able to resist the urge to gamble (from 0 – not at all to 10 – very)? | Craving self-efficacy | 5. Rating 0-10 |
| 1. Right now, how confident are you that you could limit/stop your gambling if you decided to (from 0 – not at all to 10 – very)? | Gambling self-efficacy | 6. Rating 0-10 |

*Note.* ^a^If participants respond ‘Yes’, the additional indented items were administered. The EMA comprised 5 core items and up to an additional 7 items (maximum 12 items).

### Table S3

Overview of baseline (T0), during intervention (T1), post-intervention (T2), and one-month follow-up (T3) evaluation measures and time-point assessed

| **Measure** | **T0** | **T1** | **T2** | **T3** |
| --- | --- | --- | --- | --- |
|  |  |  |  |  |
| **Acceptability** |  | | | |
| ‘Urge curbing tip and activity’ helpfulness ratings |  |  | X |  |
| EMA item relevance and burden ratings |  |  | X |  |
| Satisfaction with the Intervention (CSQ-3) |  |  | X |  |
| Impact of the Intervention (MARS App-Specific subscale) |  |  | X |  |
| Qualitative feedback: Suggested improvements for intervention content; intervention and EMA technical difficulties; general feedback |  |  | X |  |
| **Feasibility** |  |  |  |  |
| Recruitment and retention | X |  | X | X |
| EMA compliance |  | X |  |  |
| EMI compliance |  | X |  |  |
| Intervention usage: EMA-prompted use; EMI use; on demand use |  | X |  |  |
| **Preliminary Effectiveness** | | | | |
| *Primary outcomes* | | | | |
| Real-time craving intensity before and after using intervention content |  | X |  |  |
| Gambling episode at subsequent EMA |  | X |  |  |
| Gambling symptom severity (G-SAS) | X |  | X | X |
| *Secondary outcomes* |  | | | |
| Gambling craving intensity, craving self-efficacy, and gambling self-efficacy at subsequent EMA |  | X |  |  |
| Gambling cravings (G-SAS Urge subscale) | X |  | X | X |
| Total gambling frequency and expenditure (series of single items) | X |  | X | X |
| Craving self-efficacy (11-point VAS) | X |  | X | X |
| Gambling self-efficacy (11-point VAS) | X |  | X | X |
| **Descriptive and diagnostic measures** | | | | |
| Problematic gambling activity (single item) | X |  |  |  |
| Problem gambling status (PGSI) | X |  |  |  |
| Treatment goal (single item) | X |  | X | X |
| Professional help-seeking behaviour (single item) |  |  | X | X |
| Hazardous alcohol use (AUDIT-3) | X |  |  |  |
| Psychological distress (distress thermometer) | X |  |  |  |
| *Note*. T0, T2, T3: Measures administered via structured online questionnaires. T1: Measures administered via the smartphone app during the five-week baseline and intervention period. | | | | |

# Figures


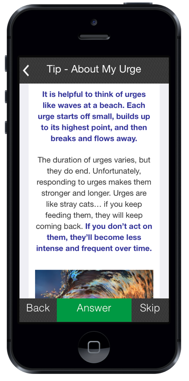

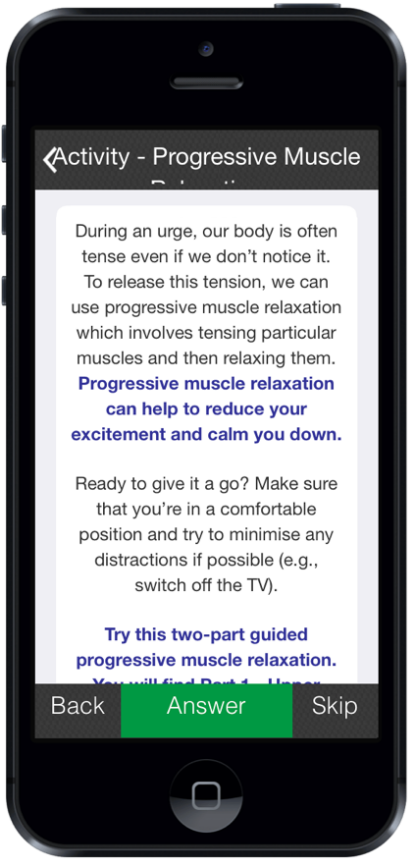

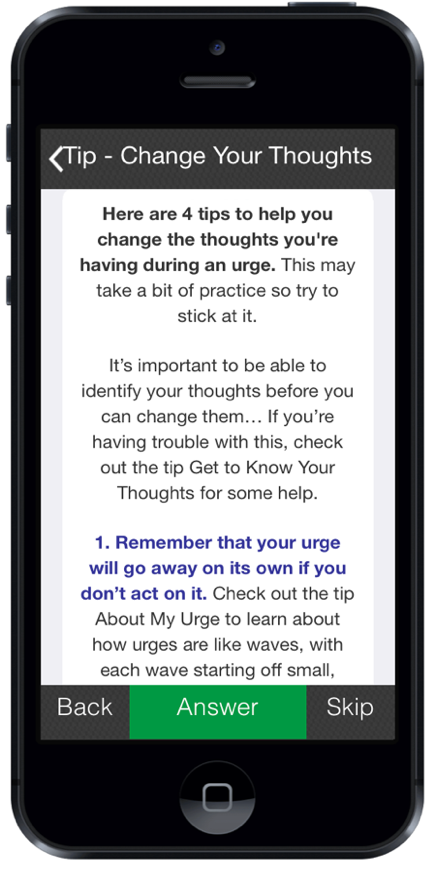


### Figure S1

GamblingLess: Curb Your Urge: Examples of ‘urge curbing tips and activities’, including About My Urge, Progressive Muscle Relaxation, and Change Your Thoughts (from left to right).

**
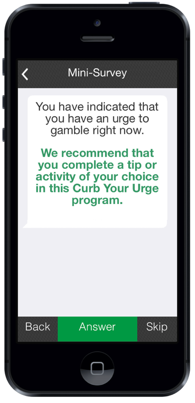
**

### Figure S2

GamblingLess: Curb Your Urge: EMI feature automatically administered at the end of any EMA where participants reported a current craving to gamble
